# BODIPY- and Porphyrin-Based Sensors for Recognition of Amino Acids and Their Derivatives

**DOI:** 10.3390/molecules25194523

**Published:** 2020-10-02

**Authors:** Marco Farinone, Karolina Urbańska, Miłosz Pawlicki

**Affiliations:** Wydział Chemii, Uniwersytet Wrocławski, F. Joliot-Curie 14, 50-383 Wrocław, Poland; marco.farinone@chem.uni.wroc.pl (M.F.); karolina.urbanska@chem.uni.wroc.pl (K.U.)

**Keywords:** molecular recognition, covalent recognition, chiral discrimination, BODIPY, porphyrin tweezers

## Abstract

Molecular recognition is a specific non-covalent and frequently reversible interaction between two or more systems based on synthetically predefined character of the receptor. This phenomenon has been extensively studied over past few decades, being of particular interest to researchers due to its widespread occurrence in biological systems. In fact, a straightforward inspiration by biological systems present in living matter and based on, e.g., hydrogen bonding is easily noticeable in construction of molecular probes. A separate aspect also incorporated into the molecular recognition relies on the direct interaction between host and guest with a covalent bonding. To date, various artificial systems exhibiting molecular recognition and based on both types of interactions have been reported. Owing to their rich optoelectronic properties, chromophores constitute a broad and powerful class of receptors for a diverse range of substrates. This review focuses on BODIPY and porphyrin chromophores as probes for molecular recognition and chiral discrimination of amino acids and their derivatives.

## 1. Introduction

For several reasons, the visible light became an important aspect of research focusing on specific interactions with the matter, which can find a wide scope of applications. The inexhaustible access to visible light opens many paths for searching for cost-efficient light-activated catalysts or systems properly interacting with specific wavelength (λ), essential for optoelectronic applications. The generality of the light also drives the activity towards formation of systems that can involve, e.g., intramolecular transformation leading to substantial change in absorbance or emission indicating a presence of specific factor. The analytical tools based on application of the visible light are constantly explored because of straightforward detection of the change even with the eye. The construction of skeleton responding to specific factors requires precise planning and designing of structural motifs that would react with and eventually modify the observed outcome, starting to behave as a molecular probe. Covalent recognition is the ability of designed molecular probes to react towards target skeletons with the formation of a covalent bond. As a result, a different behaviour, especially the optical response, can be used to monitor the presence of the target molecule in the medium that is tested. Molecular recognition is a specific tendency of compounds to selectively bind to each other through non-covalent interactions [1,2,3], such as metal coordination [4], hydrogen bonding [5], hydrophobic interactions [6], van der Waals interactions [7], π–π stacking [8] or electrostatic interactions [9]. The term “chiral recognition” refers to an event of molecular recognition, in which a receptor exhibits higher affinity towards one of existing enantiomers of guest molecule. If the host molecule is chiral, the formed host-guest complexes are diastereoisomeric and therefore possess sets of different properties, enabling their easy separation, as well as differentiation in ^1^H NMR spectroscopy (chiral discrimination). 

**Scheme 1:**
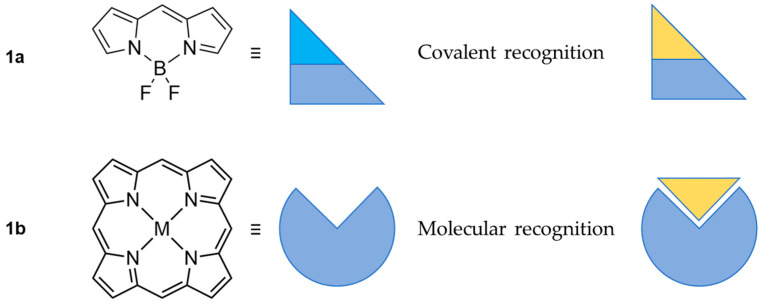
Representation of the difference in the target recognition between: BODIPY probe (**1a**); and [18]Tetraphyrin(1.1.1.1) probe (**1b**) (*porphyrin*). The yellow triangle represents the target molecule, the light blue triangle represents a leaving group.

In the past decades the BODIPY chromophores (Scheme 1, top) as well as porphyrins (Scheme 1, bottom) or porphyrinoids have found widespread applications as sensors for detection of a broad scope of substrates based on several straightforward detection channels, including UV/Vis, fluorescence and circular dichroism (CD) spectroscopies. These techniques provide a quick and accurate response in addition to requiring minimal amounts of analyte. It usually needs a properly defined optic response followed by high extinction coefficients and effective fluorescence with high quantum yield. The linear construction of BODIPY leads to sharp absorption and highly efficient emission observed, what opens a potential for application of those skeletons as optical probes. On the other hand, owing to the unique properties of porphyrinoids, they are interesting candidates for molecular [10] and chiral [11] recognition with multi-channel and straightforward UV/Vis, fluorescence and CD detection [12,13]. The macrocyclic character of porphyrin (Scheme 1, bottom) shows another aspect of oligopyrroles. Strongly conjugated π-clouds with a diatropic circuit operating within the porphyrins 18π electron path can be also applied in NMR detection, eventually showing a significant difference in registered chemical shifts. On the other hand, their rich optical properties, such as intense absorption in the Soret region (400–430 nm) and fluorescence, gives further possibilities for using them as probes as their strong and narrow absorption and fluorescence bands allow detecting even a slight perturbation caused by a guest binding.

The abundance of molecular recognition phenomena in living organisms [14] is one of the many reasons this field of interest draws attention of many researchers. Herein, we present recent reports on BODIPY- and porphyrin-based sensors for the recognition of amino acids and discuss their respective applications. The importance behind the recognition of amino acids lies on the fact that those molecules are essential in the human body for many physiological processes such as protein synthesis, detoxification, metal binding and many more [15]. The over- or under-expression of those natural derivatives is usually strictly related to different pathologies, hence the ability to detect such abnormalities is very useful especially for the screening of diseases in patients. Moreover, recognition of amino acids and other natural derivatives is a powerful tool that, if combined to the peculiar optical properties of BODIPY molecules [16,17], finds applications in bioimaging which is a non-invasive diagnostic tool of extreme importance [18]. 

The detection of other natural derivatives based on amino acids, such as G-proteins [19], neurofibrillary tangles [20] and bio-thiols [21], are strongly related with many cellular functions and pathologies and are also mentioned in this work. 

## 2. BODIPY Probes

**Scheme 2:**
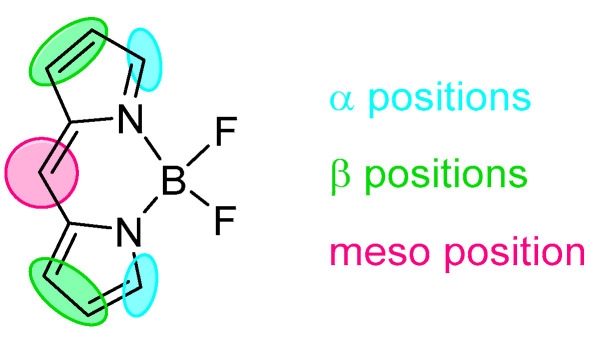
Examples of possible modification on the BODIPY core.

BODIPY (BOron(III) DIPYrromethene) is a class of chromophore formed from two pyrrolic units linked by a methylene bridge (Scheme 1 and Scheme 2) in addition to coordinating electron-deficient boron(III). The planarity of those molecules jointly with the presence boron(III) give structures with extraordinary optical properties that are fascinating the research world for many decades. Extensive investigation and studies on the synthetic aspects of those derivatives have been carried out and allowed the design of many chromophores with versatile electronic properties. Those skeletons have found applications as probes since they have a peculiar optical behaviour presenting very sharp emission bands with a quantum yield close to a unit and more importantly even small changes in the molecular structure can trigger drastic shifts in the fluorescence spectrum [22,23,24]. Because of this, BODIPY probes are widely used as molecular recognisers for the target with covalent interaction; however, the involvement of non-covalent interaction was also reported as an efficient process allowing efficient recognition. The most important features of those molecules rely on the fact that they present narrow absorption and emission bands together with high quantum yields approaching a quantitative fluorescence [25] forming skeletons potentially applicable in several fields including the recognition of specific targets. The BODIPY core was extensively explored with an involvement of specific positions (Scheme 2), eventually leading to extraordinary behaviour including tuning of the optical properties with a modulation of absorption and emission wavelength in both directions–bathochromically [26] and hypsochromically [22]. Most of the modifications showing the skeletal dependence of the optical properties are based on covalent interaction but the probing behaviour needs a response to specific initiator or target potentially available with the applied structure.

One of the most fascinating ability for those chromophores is the change in the optical response triggered by simple initiators such us pH, presence of anions and other factors [27]. Because of this behaviour, BODIPY molecules are very useful optical tools, finding many applications including the probing of, e.g., metals [28] or nitrate [29]. One other field where BODIPY derivatives are involved is the bioimaging and detection of specific targets found in the human body for many different reasons and applications [18].

The detection of those components can be achieved in different ways but, in general, a shift or the ON/OFF switch of absorption/emission is the final outcome in the recognition of a target.

**Scheme 3:**
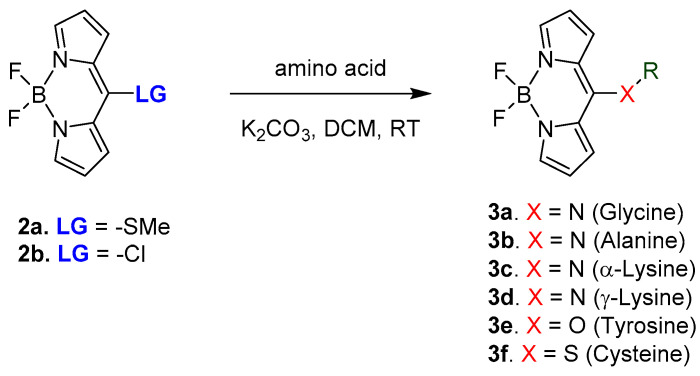
The amino acids used in this work are alanine, glycine, lysine, *p*-amino-phenylalanine, tyrosine and cysteine.

A recent work published by Farinone et al. in 2020 showing the optical behaviour for BODIPY derivatives connected to different amino acids is the example to introduce the topic of BODIPY-amino acid derivatives [23]. The hybrid derivatives were obtained through nucleophilic aromatic substitution in the *meso-*position of the BODIPY chromophore, as shown in Scheme 3.

Based on the type of heteroatom involved in the *meso* bond (**3x**), hence what type of amino acid was used, a difference in the electronic properties was recorded with shifts in both emission (range, λ_max_ = 452–535 nm) and absorption (range, λ_max_ = 407–519 nm). The most red-shifted for both absorption and emission was observed for **3f** and the biggest hipsochromic shift was recorded for **3d**. Those observations show how the optical properties of BODIPY skeleton can be modulated by a simple modification making this chromophore a versatile example of the optical probe.

### 2.1. Covalent Interaction (Recognition)

**Scheme 4:**
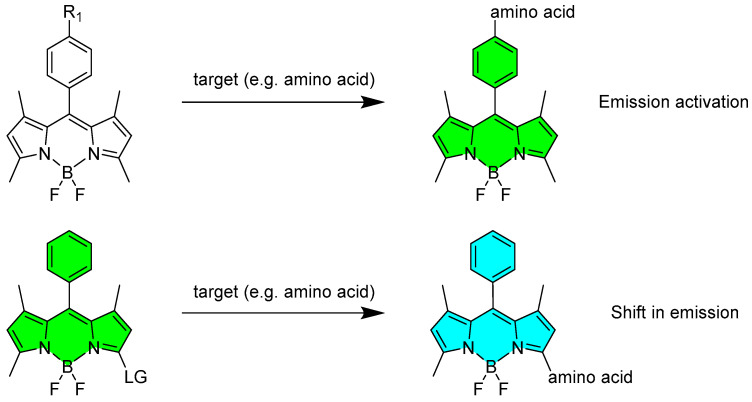
Representation of the two approaches in the recognition through covalent interaction observed for BODIPY chromophore. R_1_, group with fluorescence quenching properties; LG, leaving group such as -Cl or -SMe.

The sensing of the target molecules can be achieved towards the formation of a direct bond between the target and the BODIPY sensor with involvement of different positions of the fluorophore core and usually follows the chemistry of a nucleophilic aromatic substitution. It usually requires a precise designing of the mother skeleton and specific location of good leaving group either at *meso-* or *α-position*. After the substitution (Scheme 3 and Scheme 4), a novel BODIPY-target hybrid structure is obtained with optical properties strongly different from the starting materials making the detection of the target molecule easy and reliable. This type of interaction between the probe and the target can trigger different type of responses, which were divided into two main categories: the activation in the emission of the probe (or, in some cases, the deactivation in the probe’s fluorescence) and the shift in emission and absorption of the probe (Scheme 4).

#### 2.1.1. Emission Triggering

**Scheme 5:**
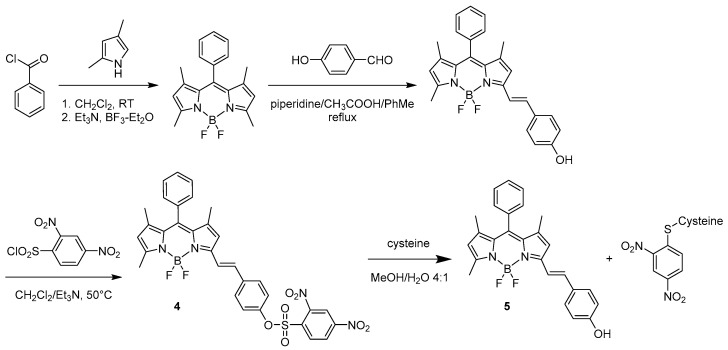
Preparation of the BODIPY probe specific for cysteine and recognition.

Depending on the planned recognition, specific response modulating optic properties are required. The potential application to biological tissues very often requires a long emission wavelength available for specifically modified BODIPY skeletons. In 2011, Shao et al. reported on a BODIPY-based molecular probe with specific detection of cysteine among the biological thiols [30]. The structure of the chromophore was designed as a sensor **4** (see Scheme 5) with a 2,4-dinitrobenzenesulfonyl (DNBS) protecting group connected to the phenol ring present on the *α* position of the BODIPY through an ethylene bridge giving a mute in emission skeleton **4**.

The recognition of the target molecule results in the unmasking of the structure **4** and eventually an appearance of intense fluorescence in the red region at 590 nm [30]. The derivative **5** presents a red shifted emission because of the specific extension of delocalisation along the *α* position.

**Scheme 6:**
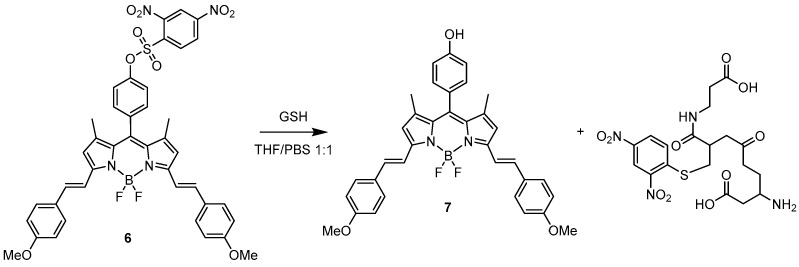
Recognition of GSH from the BODIPY probe with deprotection of quenching group.

Another example based on the enhancement of the emission toward the elimination of a protecting group with quenching properties was reported by Wang et al. in 2018 [31]. They designed a BODIPY probe (**6**) (Scheme 6) for the specific recognition of glutathione (GSH). The sensor was functionalised with a 2,4-dinitrobenzenesulphonyl group as a fluorescent quencher but more importantly as a good leaving group reactive toward thiols. As expected, **6** presents very low emission (with a quantum yield of *Φ* = 0.03) at 656 nm. The probing behaviour of **6** was tested in a THF/PBS 1:1 solution by reaction with various natural amino acids: lysine, asparagine, histidine, phenylalanine, tryptophan, glutamic acid, asparagine and cysteine, as well as homocysteine and glutathione. Cys and Hcy enhanced the fluorescence response with a quantum yield reaching 24% and 12%, respectively, at 659 and 657 nm while with GSH it reached 48% at 656 nm. Considering the differences in the intracellular concentrations of those natural derivatives, the response of the probe is remarkably selective toward GSH when tested on MCF-7 cells. All other amino acids did not affect the efficiency of the fluorescence emission.

The mechanism of recognition of GSH by the BODIPY probe is shown in Scheme 6 displaying the deprotection of the 2,4-dinitrobenzenesulphonyl moiety.

A covalent interaction between the BODIPY probe and the target molecules resulting in the activation of the chromophore emission was also presented in 2017 by Wang et al. [32]. In this work, they prepared a probe that can distinguish cysteine and homocysteine over GSH. They designed and synthetised a probe (**8**) featuring *p*-aminophenylthio and cyano groups attached to the BODIPY moiety. The decision on the presence of those groups was inspired by previous documented works that pointed out how specifically a *p*-aminophenylthio moiety can detect natural thiols.

**Scheme 7:**
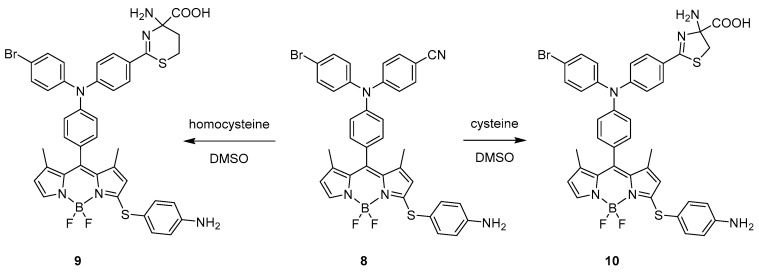
Proposed mechanism for the recognition of cysteine and homocysteine by BODIPY-based probe.

The free probe presents an absorption peak at 546 nm with substantially diminished fluorescence. The presence of sulphur from cysteine attached to the *meso*-position of BODIPY skeleton was reported as bathochromically shifting factor [23], but, after treatment with Cys (**10**), the absorption peak shifted hypsochromically by ~60 nm to 480 nm with a dramatic change in fluorescence efficiency eventually observed at 549 nm. The hypsochromic shift of BODIPY absorption was reported for *meso*-nitrogen substituted skeletons of chromophores [33] further shifted by deprotonation of *meso*-NH group [22]. A similar behaviour was detected in the case of Hcy with the formation of **9** (Scheme 7). In contrast, upon treatment with GSH, only a slight enhancement in the fluorescence was detected with an increase in intensity of 18% when compared to Cys and Hcy, indicating that the probe is successfully discriminating Cys and Hcy over GSH. The proposed mechanism of interaction of the BODIPY with cysteine and homocysteine (Scheme 7) incorporates formation of 5- and 6-membered rings obtained upon nucleophilic attack of the thiol groups. In the case of glutathione, the formation of such derivatives is possible but not favoured since the molecule is much bulkier when compared to the two others.

**Scheme 8:**
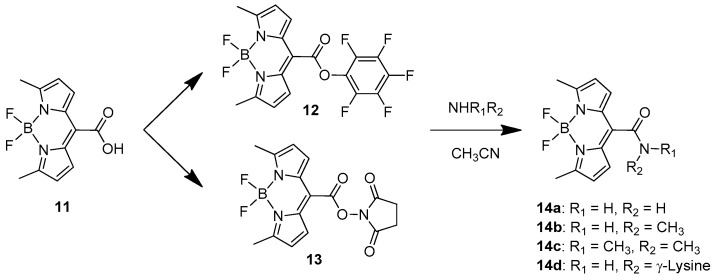
Mechanism of recognition of the amine target.

One recent example of BODIPY-based amino acid sensor was presented in 2020 by Jeon et al. [34]. They designed and developed a BODIPY sensor with high affinity for amino groups by introducing at the *meso*-position a good leaving group with an active esters type of bond well known from peptide synthesis (Scheme 8).

Two similar probes are available, one bearing a pentafluorophenyl group (**12**) and the other one with a hydroxysuccinimidyl substituent (**13**). They both present very high recognition ability toward volatile amines such as ammonia, methylamine and dimethyl amine. While **12** was demonstrated to mostly find application in detecting amines in organic solutions and their vapours in solid states, **13** was investigated in water solutions toward the recognition of biological amines such as lysine residues on proteins, antibodies and nucleic acids. The recognition tests were carried out in PBS buffer (10 mM, pH = 7.4) containing 1% acetonitrile at room temperature. The probe presented an absorption peak at 622 nm, while the emission, recorded at 648 nm, was very weak (*Φ* = 0.001) because of aggregation-caused quenching. Upon stepwise addition of l-lysine, the absorption band rises to 517 nm with a blue shift of 105 nm because of the less electron withdrawing character of the *meso* amide specie obtained (**14d**). The emission, after addition of the specific amino acid, is drastically turned on and shifted to 547 nm. A similar behaviour was observed for arginine, demonstrating that the guanidine group and the ε-amino group of lysine react very efficiently in this recognition process. Other amino acids were tested but resulted in a very weak response with longer incubation times for proline, histidine and cysteine while for the other amino acids the response was even weaker. 

**Scheme 9:**
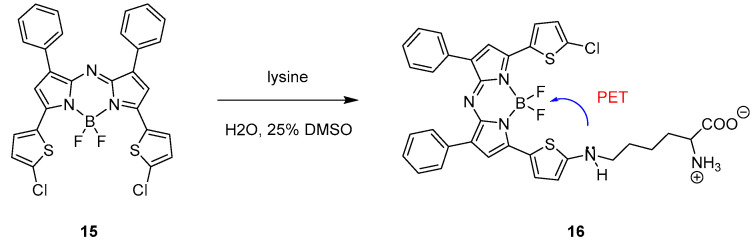
The recognition of the amino acid (lysine) shows a PET effect.

Aza-BODIPY probe **15** (Scheme 9) reported by Xiao and co-workers selectively responds to lysine with a drastic decrease of fluorescence [35].

The aza-BODIPY probe **15** presents an absorption maximum at 732 nm and a strong emission in the NIR region at 753 nm in dichloromethane. After incubating the probe with lysine in a 1:4 DMSO/water mixture (pH = 7.3), the fluorescence intensity strongly decreased because of the photo-induced electron transfer (PET) shown in Scheme 9 from structure **16**. The selectivity for the target was demonstrated testing different amino acids and bio-thiols, which, after incubation with the probe, did not show a change in the fluorescence intensity. Because of this specific behaviour, the BODIPY-based probe was eventually used in a bioimaging application to sense lysine in PC3 cells showing good and promising results.

**Scheme 10:**
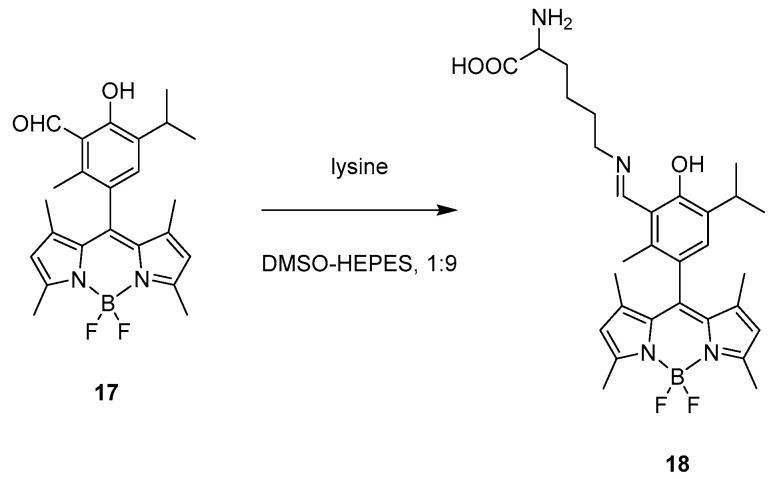
The recognition of lysine from probe **17** shows the formation of an imine bond.

A similar recognition behaviour was presented in 2016 by Das and co-workers. In their work, they synthetised a BODIPY-based probe (**17**, Scheme 10) which exhibits specific recognition abilities for lysine over other amino acids [36].

The optical behaviour of the probe consists on a sharp absorption band at 503 nm and a strong green emission with maximum at 515 nm; however, after addition of lysine in a 1:9 DMSO/HEPES buffer (pH = 7.4), absorption gradually shifts blue to 497 nm, together with the formation of a broad band at 550 nm, while emission is effectively quenched. This behaviour relies on the specific design of the probe which presents an aldehyde group highly reactive toward lysine; indeed, after addition of the amino acid, an imine bond is formed, as shown in structure **18**, Scheme 9. An ^1^H NMR titration of the probe with lysine supports the proposed mechanism of recognition with the formation of the Schiff base, the aldehyde proton is gradually decreasing and substituted by a new imine proton. Because of those characteristics, the probe was tested for bioimaging. It was incubated with MDA-MB 231 cells and showed a gradual quenching of fluorescence upon addition of lysine.

#### 2.1.2. Emission Shift

As presented above, the fluorescence on/off behaviour of specific covalent modification applied for BODIPY skeleton can be used for detecting the presence of specific analytes. Slightly different approach for detection is also possible and is based on easily detectable change in absorbance/emission wavelength triggered by interaction between probe and detected molecule (see Scheme 4).

**Scheme 11:**
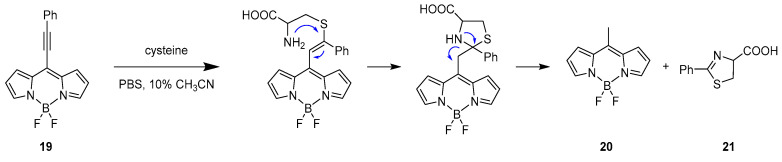
The dual Michael addition reaction proposed for this cysteine recognition [29].

One example using this specific method to selectively recognise cysteine over other bio-thiols such as homocysteine and glutathione (GSH) was reported by Liu et al. in 2015 [37]. An 8-alkynyl-BODIPY fluorescent probe was designed having an activated alkynyl unit connected to the *meso* position, **19**. The recognition of the targets follows the reactivity of dual Michael addition, as shown in Scheme 11.

The probe presents two absorption bands at 410 and 538 nm and one emission band in the yellow region at 560 nm. Upon addition of cysteine in a CH_3_CN/PBS buffer (pH = 7.4) at 25 °C, the absorption bands were replaced by a single absorbance at 489 nm, while, for the emission, a hypsochromic shift was observed to 505 nm responsible for the formation of 8-methyl-BODIPY derivative as product (**20**) of the retro-aza-aldol process triggered by the dual Michael addition.

**Scheme 12:**
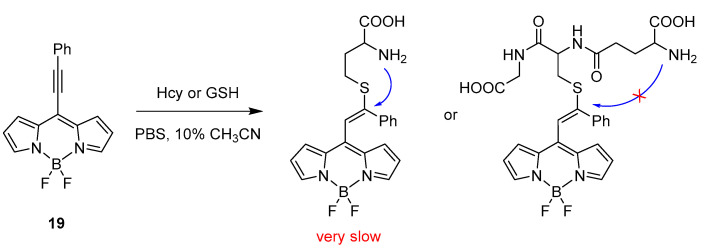
Specificity of the recognition toward cysteine is explained according to structural differences.

The specificity towards recognition of cysteine over homocysteine and glutathione is presented in Scheme 12. The structural similarities (nucleophilic thiol and amino group) in the architecture of Hcy and GSH with Cys should not preclude a cascade reaction eventually leading to an optical response. However, the limiting step for Hcy and GSH is the formation of the cyclic transition state, which will generate six- and ten-membered rings, respectively. This kinetically unfavoured step makes the cascade reaction very slow for Hcy and impossible for GSH, allowing a high specificity for cysteine.

**Figure 1:**
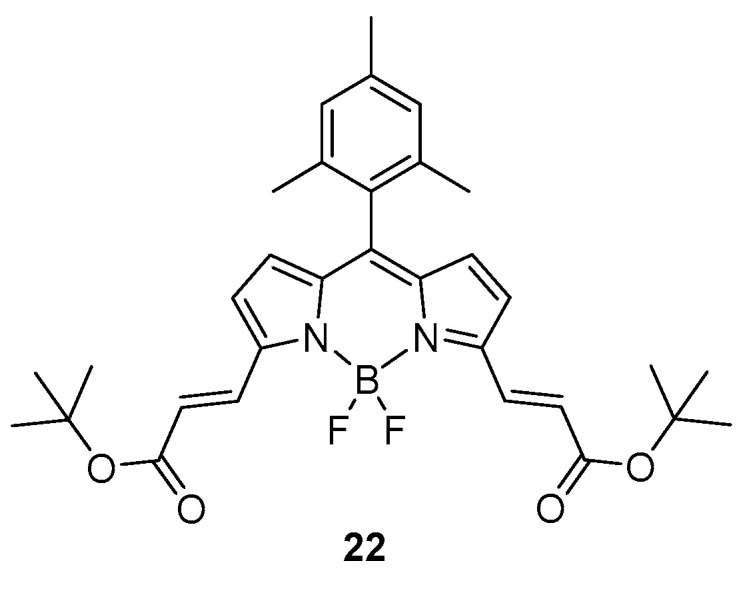
Structure of the BODIPY sensor **22** specific for detection of cysteine, homocysteine and glutathione.

In 2019, Avellanal-Zaballa et al. presented a BODIPY probe designed with functionalities responsive to thiol containing amino acids [38]. The sensor presents two *α,β*-unsaturated esters on both *α* positions of the BODIPY core (**22**, see Figure 1) as the reactive points that are able to react with the nucleophilic groups of the sensed targets. The formation of the probe was possible starting from a di-formylated derivative sequentially converted to the unsaturated ester through Wittig reaction.

Because of the extension of delocalisation through the *α* positions of the sensor, an absorption band is present at 590 nm and an emission band is recorded at 600 nm. After treatment with any of cysteine, homocysteine or glutathione, the absorption band is gradually replaced by two blue shifted peaks at 550 and 515 nm; the emission is similarly shifted to shorter wavelengths at 560 and 525 nm. The tests were carried out in a 1:1 mixture of ethanol/HEPES to mimic the physiological media. Even at low concentrations, the detection of targets (micromolar) was possible by monitoring the fluorescence; hence, the detection of bio-thiols is easily accessible using the described probe.

##### Intermolecular Displacement

In some specific cases where a shift in absorption and emission is detected upon covalent interaction between the probe and the target, the mechanism for the change in the electronic properties is based on an intermolecular displacement.

**Scheme 13:**
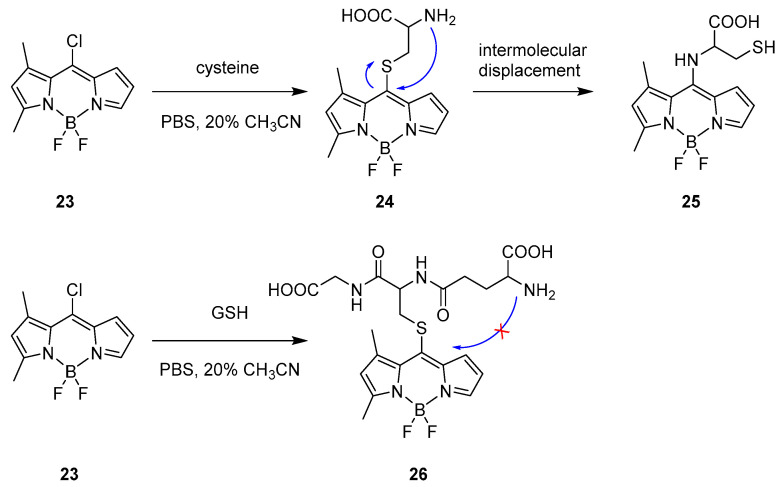
Proposed mechanism for the cysteine recognition with resulting formation if **18** and limitation over GSH recognition.

In 2018, Zhao and co-workers reported a BODIPY chromophore applicable for specific recognition for cysteine in human plasma [39]. In this case, it presented a strong emission and with no protecting groups in the architecture. The detecting approach starts from formation of cysteine derivative **24** (Scheme 13) that undergoes intermolecular transformation changing the optical behaviour with the formation of **25**.

The probe **23** was chosen because of its stability in buffer solutions and the presence of a good leaving group at the *meso* position. Moreover, the structure was designed as an asymmetric molecule with steric substituents favouring a ratiometric response. The fluorescence spectrum was recorded in a CH_3_CN/PBS buffer (pH = 7.4) at 25 °C displaying main absorption and emission at 491 and 515 nm for probe **23**. After addition of cysteine, a nucleophilic aromatic substitution took place in the *meso*-position and a ratiometric response was observed with a dramatic shift both on absorption and emission with maxima recorded at 412 and 470 nm. The strong shift in the emission is not explainable with the presence of a sulphur at the meso position; according to other reports presenting dissimilar properties [23,33]. Indeed, due to the intramolecular displacement described in Scheme 13, the amino group replaced the sulphur function giving and explanation to the strong shift.

The specificity of this probe relies on the fact that upon treatment with another thiol rich natural derivative, glutathione (GSH), the absorption peak at 491 nm rapidly decreased and a red-shifted peak at λ_max_ = 509 nm was formed. In addition, a new emission wavelength was measured at 537 nm and both optical shifts were not ratiometric and support the formation of a thioether intermediary, **26**; indeed, the intramolecular displacement is not available for GSH, as shown on Scheme 13.

**Scheme 14:**
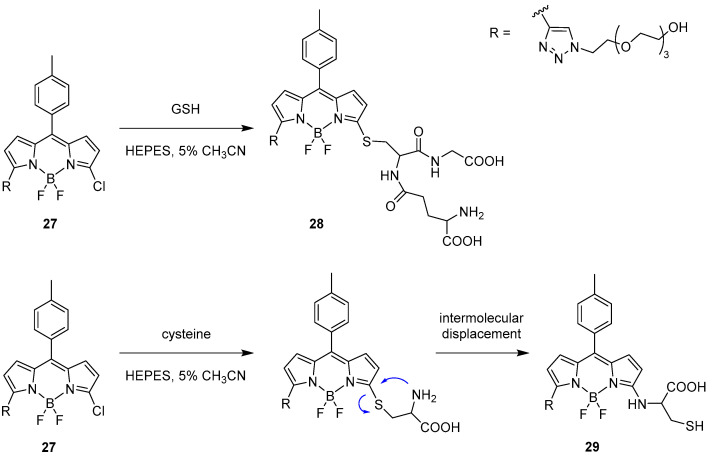
Recognition process for the BODIPY probe **25** toward GSH and proposed molecular displacement for cysteine.

Another example was presented in 2012 by Niu et al. [40]. The focus of their work was the ability to selectively detect the presence of glutathione (GSH) over cysteine and homocysteine. To achieve this, they designed a BODIPY-based probe (**27**, Scheme 14) substituted with a chlorine atom on the *α* position as a good leaving group and a triazole on the other *α* position to enhance the reactivity toward nucleophilic aromatic substitution because of the strong electron withdrawing character of this substituent. Moreover, the BODIPY derivative presents good solubility in water and a defined emission peak at 556 nm. The fluorescence response of the probe was tested in an aqueous HEPES buffer (20 mM, pH = 7.4) containing 5% acetonitrile at 37 °C with stepwise addition of GSH. As a result, the λ_em_ = 556 nm emission peak was gradually replaced by a new band at 588 nm with a fluorescence ratio proportional to the concentration of GSH and to the formation of derivative **28**.

The behaviour of the probe was tested with other bio-thiols such as cysteine and homocysteine. With Cys, after the initial addition of the nucleophile, a new emission band appeared at around 590 nm but rapidly decreased, favouring another band at 564 nm according to the proposed intermolecular displacement shown in Scheme 12 and resulting in the formation of **29**. In case of Hcy, similar to GSH, an emission band at 587 nm appeared but it was followed by a strong decrease in the intensity, following the same reactivity as proposed for cysteine.

### 2.2. Molecular Recognition (Non-Covalent)

A different approach for the recognition by BODIPY probes can be used especially for the detection of amino acids and small peptides. For example, precise functional groups can be placed in the BODIPY architecture so that it will specifically interact in a non-covalent fashion with the target molecule, eventually resulting in a change of the spectral profiles. Few reports following this mechanism have been published for BODIPY probes and amino acid derivatives, and it is possible to find examples with other natural derivative such as G-proteins. 

**Figure 2:**
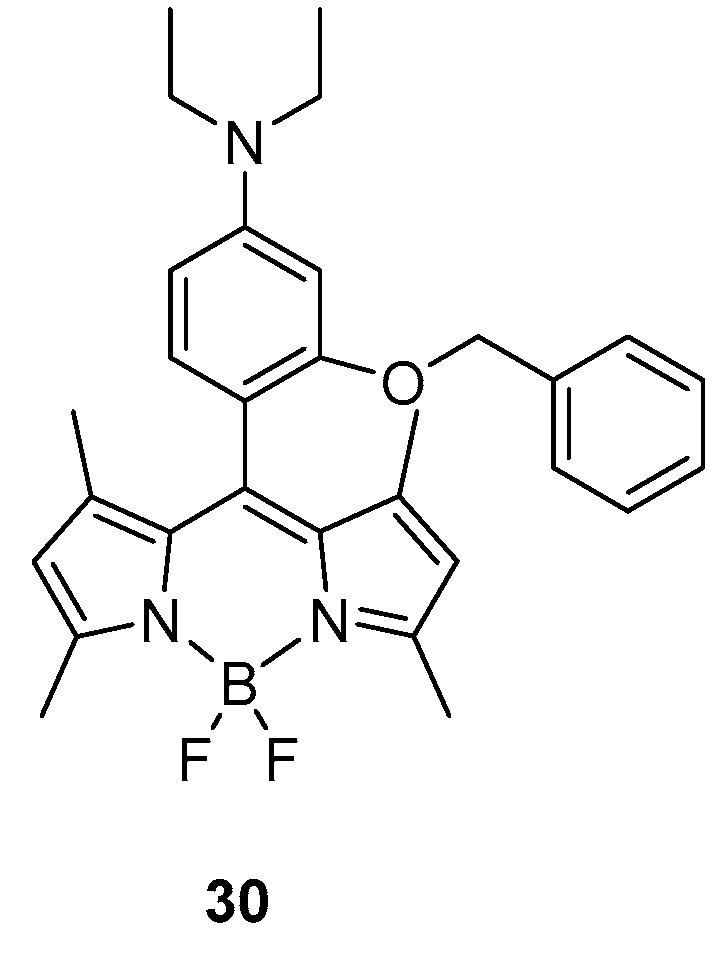
The BODIPY sensor that presents a photo-induced electron transfer (PET) process in water.

One interesting work focused on the rapid detection of aspartic acid and glutamic acid in water was recently reported by Guria et al. [41]. In their approach, a BODIPY probe was designed with a specific architecture bearing a diethyl amino substituted moiety connected to the *meso* position, as shown in Figure 2, **30**.

Because of the specific construction of the probe, a photo-induced electron transfer (PET) is observed with only a very weak emission detectable at 527 nm in water. Upon addition of aspartic acid (Asp) or glutamic acid (Glu) (0–600 μM amino acid, 3:97 DMSO/H_2_O, pH 6.5), the emission maximum was recorded to rapidly increase as a consequence of the interaction between the probe and the amino acid, which is effectively blocking the PET process. In addition, the absorbance was strongly influenced upon addition of the target molecule; the main absorption band at 514 nm was hypsochromically shifted to 503 nm. The type of interaction between the probe and the target molecule was ascribed to be of hydrogen bonding with theoretical (DFT) and NMR evidence; indeed, significant changes in the chemical shifts of the protons in the pyridine moiety were detected.

The highly selectivity of the system for Asp and Glu was demonstrated by comparing the behaviour with all the other amino acids that, when added to the probe, did not significantly alter the fluorescent profile of the BODIPY. Because of those specific behaviours, the probe was used for intracellular imaging and showed good response and high stability in the cellular systems.

**Figure 3:**
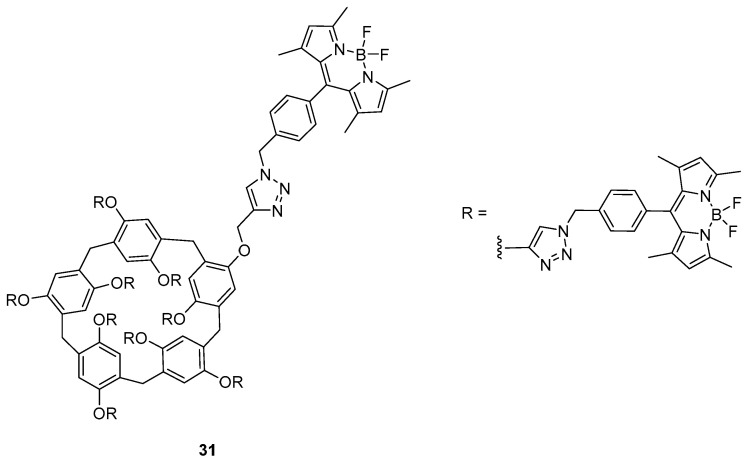
The pillar [5]arene BODIPY sensor presented in Guler’s work.

Recently, Guler and co-workers presented an interesting work about a BODIPY probe based on a pillar[5]arene structure with sensing ability for asparagine. The probe (**31** in Figure 3) was obtained by click chemistry between an azido BODIPY precursor and a pillar[5]arene having terminal alkynes [42].

The cyclic structure presents absorption and emission maxima, respectively, at 325, 505 and 520 nm. Upon addition of thirty different amino acids and derivatives in a DMF/H_2_O 1:1 mixture, the only appreciable changes were recorded in the emission spectrum when l-asparagine was used. Specifically, the emission intensity was enhanced and a slight blue shift of 6 nm was recorded. The type of interaction between the probe and the sensed specie was described from the authors to be non-covalent with the formation of an inclusion type of complex. The host-guest nature of interaction is supported by NMR experiments, which show how all the protons of the amino acid are reasonably upfield shifted because of the rich electron environment of the cavity.

**Figure 4:**
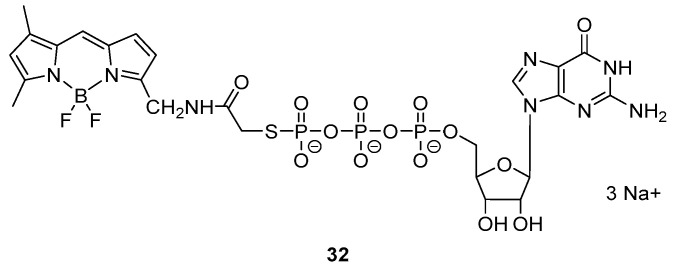
The GDP–BODIPY hybrid sensor investigated in Kennedy’s work.

Kennedy et al. designed and synthetized a GDP analogue integrating a BODIPY moiety (**32**) (Figure 4) which presents a fluorescence emission with maximum at 510 nm [19].

Interestingly, the incubation of the probe with G-protein’s *α* subunits in a Tris buffer (192 mM glycine, 1 mM EDTA, 10 mM CaCl_2_, pH = 8.5) resulted in a fluorescence anisotropy, indicating the formation of a complex. As control, the probe was tested with trypsin inhibitor and no change in anisotropy was noticeable.

**Figure 5:**
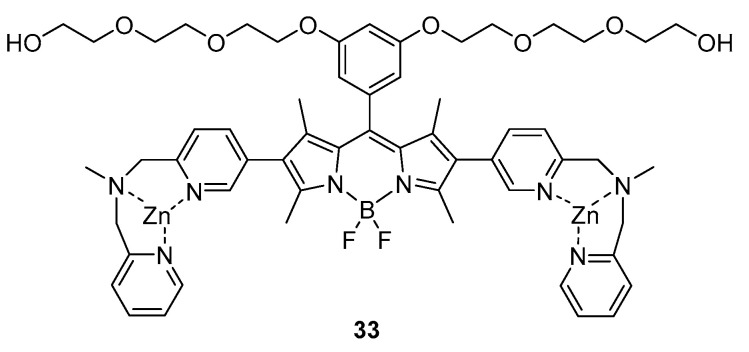
BODIPY probe with bipyridyl-type Zn(II) complex moieties.

Another example reporting a non-covalent interaction between the probe and the target was documented in 2009 by I. Hamachi and co-workers [20]. In their work, they developed a BODIPY-based probe able to interact and identify the Neurofibrillary Tangles presents in patients affected by the Alzheimer’s disease. They designed a probe with bipyridyl-type Zn(II) complex moieties, i.e., **33**, which can easily interact with hyperphosphorylated tau proteins and that presents absorption and emission bands at 520 and 547 nm (*Φ* = 0.31), respectively (see Figure 5).

Upon addition of the probe in HBS buffer (100 nM) to a suspension of *p*-Tau (8 µM, overexpressed in Alzheimer’s disease) in HBS buffer containing 10% DMSO, an increase of intensity in the emission was recorded and a dissociation constant of 7.5 μM was calculated. When the sensor was mixed with mono- or non-phosphorylated tau, no changes in the emission could be appreciated, showing a specificity of the probe for the hyperphosphorylated tau proteins.

## 3. Porphyrin Probes

**Figure 6:**
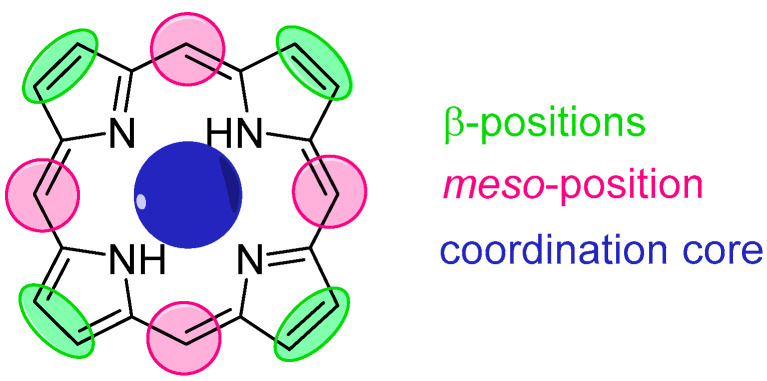
Porphyrin modification sites.

The structure of porphyrin, namely [18] tetraphyrin(1.1.1.1), provides three types of modification sites: eight β positions, four *meso* positions and the coordination cavity (Figure 6). Porphyrin hosts for amino acid derivatives typically make use of both the peripheral alterations and metal coordination, the latter being of key importance for such applications. The *meso*-substituents typically include hydrogen bond donors (e.g., hydroxyl groups) and acceptors (e.g., methoxyl groups) and bulky aromatic rings (e.g., naphthyl). Formation of stable supramolecular complexes is achieved via three-point fixation, consisting of coordination of amino acid *N*-terminus to the metal centre, as well as hydrogen bonding and electrostatic, hydrophobic or steric interactions.

Porphyrin’s N_4_ coordination cavity allows binding of numerous metal ions. A careful choice of metal centre allows coordination of axial ligands, for instance amines [43] or alcohols [44]. Such coordination causes slight perturbation to the optical properties, easily detected with UV/Vis spectroscopy. Examples of sensors described in this section involve zinc(II) and ruthenium(II) porphyrins.

**Scheme 15:**
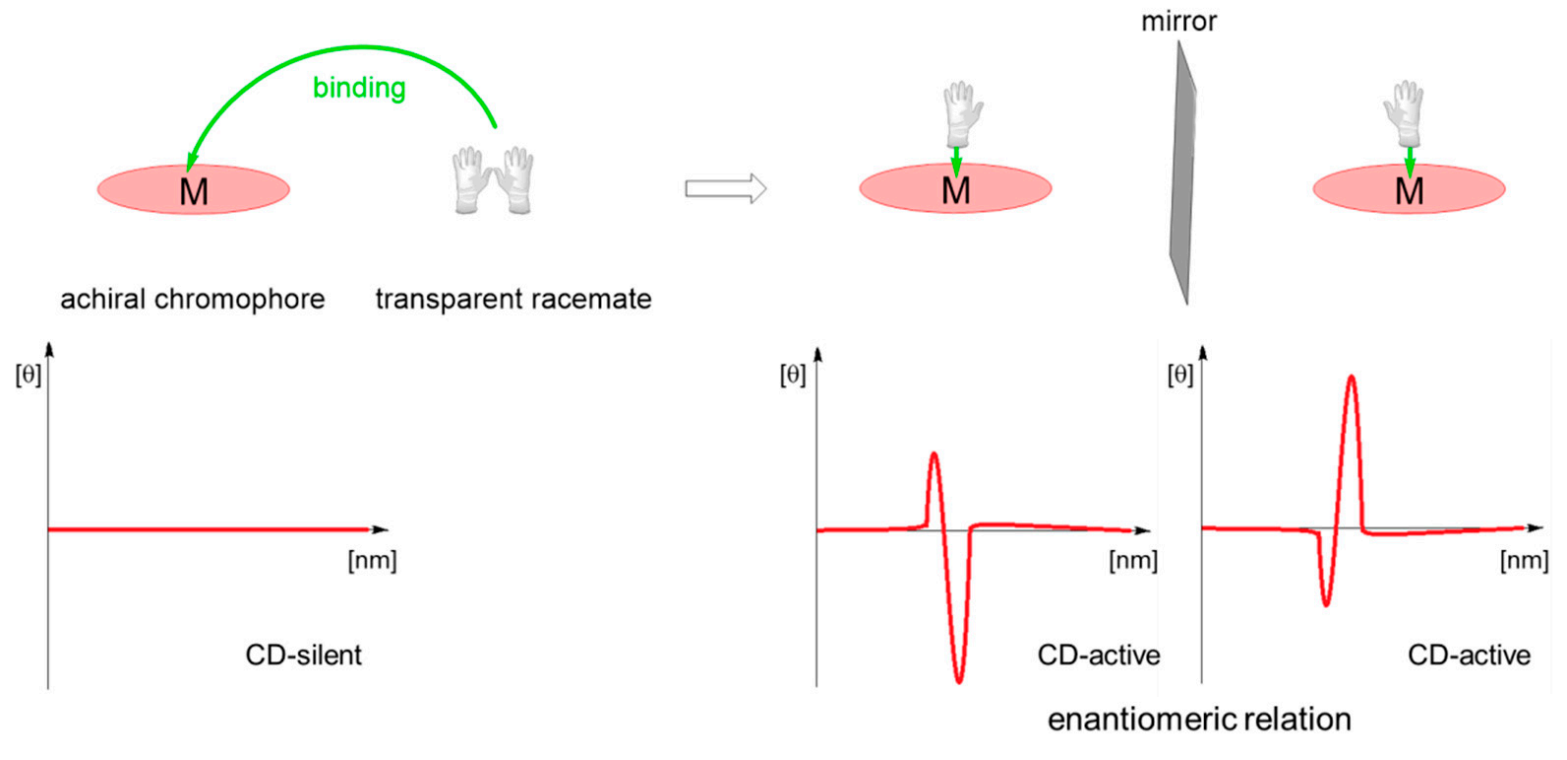
Schematic presentation of supramolecular chirogenesis in porphyrin-amino acid host-guest systems.

Binding of a chiral molecule to an achiral porphyrin results in chirogenesis-process of transfer of chiral information from the chiral partner (guest) to achiral host [45]. This leads to induction of supramolecular chirality with characteristic strong Cotton effects, carrying information about the guest absolute configuration (Scheme 15) [46]. Formation of supramolecular host-guest complexes described in this paper is therefore easily detected with circular dichroism spectroscopy.

### 3.1. Monomeric Hosts

#### 3.1.1. Early Reports

The use of porphyrins as probes for chiral recognition of amino acids dates back to the early 1990s, when Ogoshi and co-workers published a series of papers describing the design and application of several hosts employing porphyrin zinc complexes with peripheral substituents tailored to bind α-aminoester guests.

**Figure 7:**
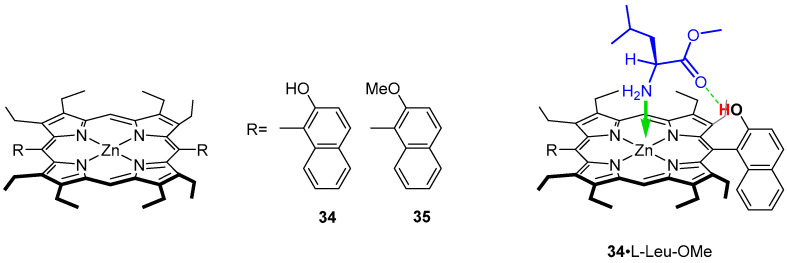
Ogoshi’s *meso*-naphthyl sensors for amino acid methyl esters.

Three hosts with various binding capability were examined (Figure 7) as zinc(II) complexes open for coordination of a guest and an auxiliary binding site: a hydrogen bond donor (**34**) and a hydrogen bond acceptor (**35**) [47]. Thus, the construction of macrocyclic host was predefined for formation of a doubly bound system with nitrogen coordinated to zinc(II) centre and a hydrogen bond interaction of a C-terminus of an amino acid. The UV/Vis titration with LeuOMe revealed formation of a 1:1 complex **34**·LeuOMe upon addition of large excess of the guest (Figure 7) manifested by the Soret band bathochromic shift treated as diagnostic of formation of host-guest system. ^1^H NMR experiments consistently support the participation of hydrogen bonding in host-guest formation. Titration of **34** with a solution of l-LeuOMe results in downfield shift of the naphthyl hydroxy proton signal (marked red in Figure 7) from δ ~5.2 ppm to δ ~6.4 ppm, indicating the presence of hydrogen bond incorporating the hydroxyl group. Further experiments performed for methyl esters of valine, phenylalanine, tryptophan, asparagine or glutamic acid showed that bulkier side chains of amino acids positively influence stability of final complexes. In addition, the presence of aromatic substituents increases the stability of final host–guest systems obtained for **35** where hydrogen bond character is inverted while carboxylic groups has no influence. The association constants for **34** (1.7 × 10^3^–1.1 × 10^4^ M^−1^) are 3.8–40.0 times larger than those obtained for **35**. These observations led to the conclusion that hydrogen bonds are formed between naphthyl hydroxyl and aminoester carbonyl groups (Figure 7).

**Figure 8:**
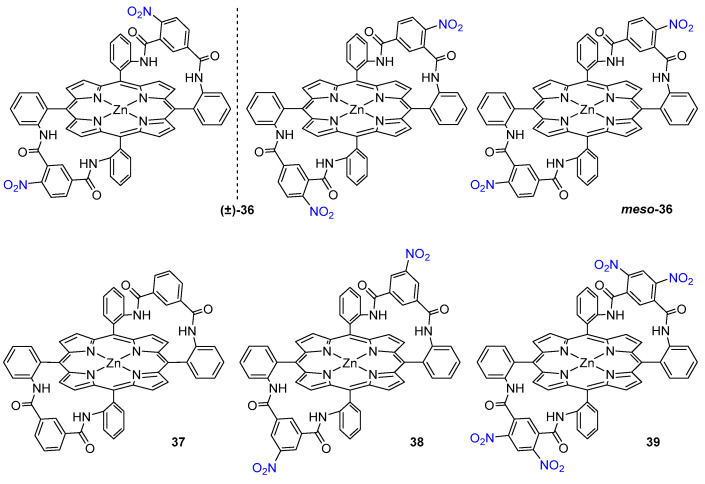
Doubly bridged chirality probes.

Ogoshi and co-workers designed also a condensed α,α,β,β-atropoisomer of *meso*-tetra(*o*-amino)phenyl porphyrin with un- and substituted isophthaloyl chlorides to yield doubly bridged chirality probes **36**–**39** (Figure 8). Both optically pure enantiomers were examined, and they exhibited significant enantioselectivity, from 4.1 for alanine to 7.5 for valine methyl ester. Upon ^1^H NMR monitored coordination experiment, all guest ^1^H signals shift upfield to negative δ values, due to the guest molecule being placed in porphyrin’s magnetic anisotropy cone [48]. In the case of l-ValOMe, the most dramatic shift difference is observed for NH_2_ and C^α^H protons (*Δδ* = −6.04 ppm and *Δδ* = −6.21 ppm, respectively) as they appear at δ = −4.57 ppm and δ = −2.90 ppm in the complex, indicating that both groups of protons come in close contact with the porphyrin plane [49].

The *O-*nitro substituents play an important role in the recognition process. It was found that **(-)-35** binds the same guests with association constants 12 and 21 times larger than **37** and **38**, indicating that the hydrogen bond donating ability of the amide group remaining in the *ortho* position to the nitro substituent is increased by the electronic effect of NO_2_ group.

#### 3.1.2. Achiral Hosts

**Figure 9:**
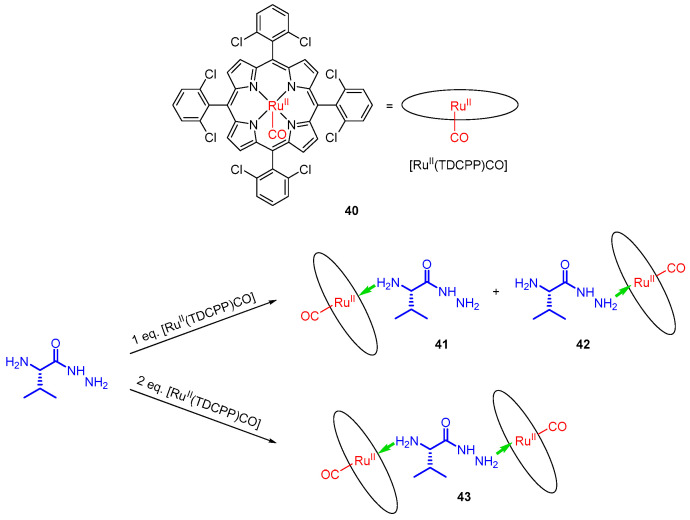
Stoichiometry-dependent host-guest complex formation.

Commercially available ruthenium(II) porphyrin complex **40**, with a strong Lewis acid character, was employed as a chirality probe for amino acid esters and hydrazides [49]. The strong binding of amines to the Ru centre is reflected in the high association constant determined for coordination of l-ValOMe as *K*_ass_ = 1.7 × 10^7^ M^−1^. The difference in coordination abilities to Ru and Zn centres is reflected by the biding constant for **40**, which is three-fold higher than those of Zn-based probes, such as **33**. Amino acid hydrazides were obtained upon hydrazinolysis of corresponding amino acid methyl esters, and their coordination to **40** was monitored with ^1^H NMR. Exposing such hydrazide to 1 eq. of **40** yields two 1:1 complexes, **41** and **42**, differing by the donor for coordination to ruthenium. Further addition to the macrocyclic host leads to formation of sandwich complex **43**, in which the two terminal amino groups coordinate to ruthenium centres (Figure 9). In such magnetic environment, all ^1^H signals of the guest move to negative values of chemical shifts (from δ = −0.5 ppm to δ = −6.4 ppm). Protons attached to the two terminal amino groups show substantial upfield shifting to δ = −2.8 ppm and δ = −3.8 ppm (hydrazide NH_2_) and δ = −5.4 ppm and δ = −6.4 ppm (C^α^-NH_2_).

The presence of chiral guest results in formation of a sandwich complex that exhibits a distinct CD effect in the Soret region (405 and 412 nm). When l isomers were used (valine and phenylalanine), the shorter wavelength band was negative and the other positive, while with d-phenylalanine derivative the CD image was reversed. Some other commercially available ruthenium(II) porphyrins ([Ru^II^(TPP)CO], [Ru^II^(TMP)CO] and [Ru^II^(F20-TPP)CO]) were tested as potential chirality probes and gave corresponding results [49].

#### 3.1.3. Chiral Hosts

**Figure 10:**
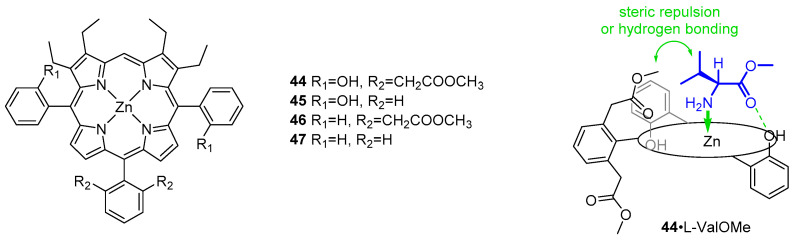
Three-point fixation of aminoesters in the cavity of porphyrin host.

Continuing their research on thermodynamic aspects of host–guest interactions between porphyrins and amino acid esters, Ogoshi and co-workers designed a set of chiral hosts based on a zinc(II) coordination centre and auxiliary hydroxyl groups used as hydrogen bond donors (Figure 10). Methyl acetate groups were introduced for a steric repulsion and hydrogen bond acceptance [50]. While the combination of coordination and hydrogen bonding in the positive isomer gave results corresponding to those reported previously for **35** [47], the presence of ester groups in **44** resulted in slightly repulsive behaviour towards the side chain of l-aminoesters. The effect was even more pronounced in a corresponding series of d-aminoesters. A significant exception was observed for d-SerOBz where the carbonyl group of methyl acetate acted as a hydrogen bond acceptor for serine side chain hydroxyl. Comparing association constants for binding of l- and d-aminoesters, it was concluded that **(+)-44** shows significant enantioselectivity for l-aminoesters, except for d-SerOBz, where the enantioselectivity is reversed, probably due to the noteworthy contribution from a second hydrogen bond. The extent of steric repulsion towards l-aminoesters was independent of hydrogen bonding, but in the case of d-aminoesters the repulsion was larger in complexes of **44** (complexes with two-point fixation, i.e., coordination and hydrogen bonding) than **45**, where no hydrogen bonding occurs (one-point fixation by coordination). When d isomers were used, the interaction energies were sensitive to the character (size and functional groups) of the guest.

**Figure 11:**
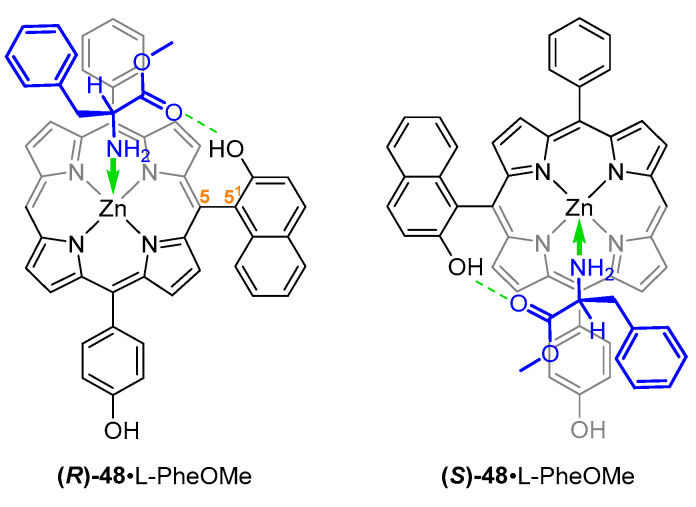
Supramolecular complexes of isomers of ABCD-type porphyrin sensor and l-phenylalanine methyl ester.

2-Hydroxynaphthyl substituent was also employed in the design of a chiral ABCD-type porphyrin reported by Yang et al. Steric hindrance of 2-hydroxyl group and 9-hydrogen atom prevents rotation along the 5–5^1^ bond (Figure 11). Enantioselectivity of **(*R*)-48** was 1.6, favouring l-PheOMe over its chiral counterpart.

The phenomenon of chiral discrimination of porphyrin-aminoester pairs was employed for NMR-assisted configuration assignment, where phenylalanine methyl esters were used as chiral solvating agents. Formation of host–guest complexes with two-point fixation via NH_2_-Zn coordination and C=O···HO hydrogen bonding led to decrease in freedom of motion of the host molecules, resulting in resolution of NMR signals in thus obtained diastereoisomeric complexes [51].

### 3.2. Dimeric Hosts

Due to their 18π electron circuit and ability to accommodate various metal ions inside their cavity, porphyrins have been used as an attractive building block for molecular tweezers [52]. A molecular tweezer is a host molecule consisting of two, usually flat and aromatic subunits, designed to “recognise” specific guest molecules by means of metal coordination, π–π interactions, hydrogen bonding, hydrophobic interactions or van der Waals forces. Such subunits are connected via a linker, which can be a spacer of greater or smaller flexibility. Since the first synthesis of a caffeine dimer with diyne linker was reported in 1978 by Chen and Whitlock [53], many molecular tweezers were designed to serve various purposes. Zinc complexes of porphyrins tend to bind neutral ligands through nitrogen, oxygen, phosphorus and sulphur, forming five-coordinated 1:1 adducts [54]. This property has been widely exploited in porphyrin tweezer applications, including those designed for chiral recognition.

**Figure 12:**
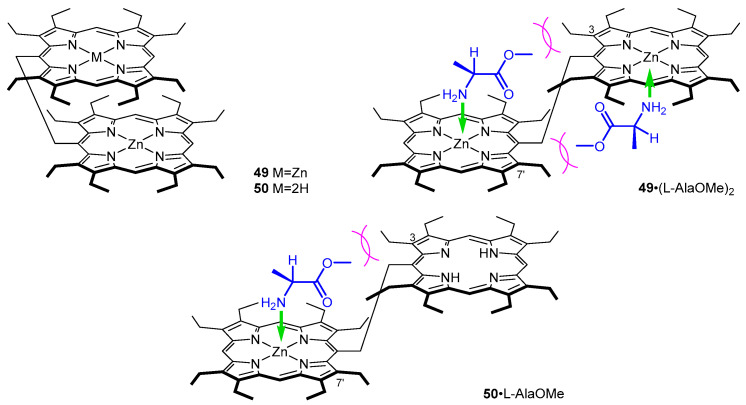
Achiral ethane-bridged *syn*-*bis*-porphyrins and their interaction with aminoester guests.

In the early 2000s Borovkov et al. reported a chirality induction in achiral ethane-bridged *syn*-*bis*-porphyrins **49**–**50** via an interaction with chiral amines and amino acids. Based on the reported dependence of CD signal sign and intensity on the steric bulk of aminoester side chain, they suggested that coordination of the enantiopure ligand induced such a transformation, where the achiral *syn* conformer **49** (Figure 12) effectively transforms into a chiral variant **49**·(l-AlaOMe)_2_. In a subsequent study, the mechanism of chirality induction was investigated and tweezers with one or two coordinating centres were employed (Figure 12) [55].

In nonpolar solvents, **49** adapts a cofacial, *syn* conformation, due to strong π–π interactions between the two porphyrin rings. Depending on the number of coordination sites, two different *anti*-conformers were observed: 1:2 for two zinc(II) centres and 1:1 for single coordination of zinc(II) as documented spetroscopically.

In UV/Vis, host–guest complexes of **49** and amino acid esters show bathochromic shift of Soret band, as well as its splitting (at 435–437 and 424–426 nm), and a set of two Q bands, among which the higher energy one is significantly more intense than the other. In CD, these systems show strong bisignate couplets in the Soret region—with maxima corresponding to those observed in UV/Vis—and an additional blue-shifted lower intensity Cotton effect.

The factors influencing the handedness of the screw are as follows: right-handed screw is formed when the C-terminus substituent is bulkier than amino acid side chain; in the opposite relation, such arrangement is unfavourable and left-handed screw is induced. Therefore, it was found that the CD response depends not only on the absolute configuration of amino acid guest, but also on the mutual steric relation between the C^α^ substituents. Thus, the Cotton effects in the complex only correspond to the guest absolute configuration when the side chain is less bulky than the COOR group. Among l isomers, positive chirality corresponds to AlaOMe, IleOMe, ValOMe, ProOMe and ProOBn and negative sign was observed in the case of LeuOMe, LeuOBn, PheOMe, PheOBn, PhgOMe, TyrOBn, Tyr(Bn)OMe, Glu(OMe)OMe, GlnO*t*Bu, CysOMe, Lys(Z)OMe and TrpOMe.

Investigation of a 1:1 host-guest complex **50**·l-AlaOMe (Figure 12) provided insight into the mechanism of chirality induction in such supramolecular complexes. It was demonstrated that steric interaction of 3- and 7′-ethyl groups in **49** and **50** with aminoester guests is the driving force behind chirogenesis (Figure 12), as it locks guest molecule in position. It was also found that the intensity of induced Cotton effects depends on the number of possible chirality-generating conformations, which increases with the number of accommodated aminoester molecules. Moreover, **50** is a suitable host for chiral recognition of bidentate guests, i.e., chiral diamines or amino alcohols, which form 1:1 CD-silent complexes with **49**. For example, a 1:1 complex of **49** and 1,2-DACH (1,2-diaminocyclohexane) is so strong, that 1:2 cannot be formed even upon addition of large excess of ligand (K_assoc_ > 10^7^ M^−1^).

**Figure 13:**
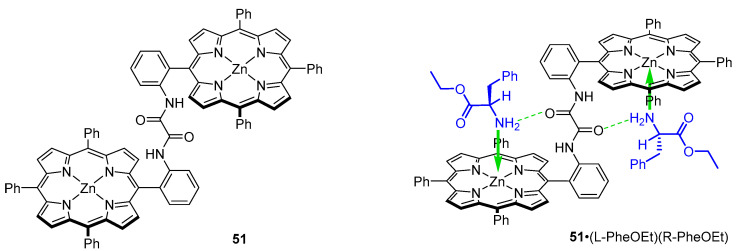
Porphyrin tweezer with oxalic amide linker and its 1:2 complex with an aminoester guest.

Jiang et al. designed three achiral Zn(II) triphenylporphyrin dimers with amide linkers of various rigidity: **51**–**53** for the purpose of elucidating the mechanism of chiral recognition in systems with two point fixation. First, oxalic amide-linked tweezer **51** was investigated as a host for nonpolar amino acid ethyl esters. Crystal structure of a 1:2 complex of **51** and *rac*-PheOEt was solved and the following structural features were brought to light: With a linker of this flexibility, the dimer adapts an *anti*-configuration in the complex, and both ligand molecules coordinate to zinc from inside the tweezer cavity. Contrary to prior reports, it was found that in **51·**(l-PheOEt)(d-PheOEt) hydrogen bonds are formed between aminoester NH_2_ and linker carbonyl group (Figure 13). For comparison, in their account on *meso*-naphthyl probe **34**, Ogoshi and co-workers suggested that the naphthyl OH group acts as a hydrogen bond donor, and amino acid carbonyl group oxygen is the acceptor (Figure 7) [48].

Formation of 1:2 complexes with **51** was documented by UV/Vis titration with solutions of amino acid ethyl esters. Two sets of isosbestic points were detected at 426 and 424 nm. K_2_ was reported as significantly lower than K_1_ for all guests employed (K_1_: 2.0–7.2 × 10^4^ M^−1^; K_2_: 1.0–1.2 × 10^4^ M^−1^), consistently with more difficult biding of the second guest due to the different coordination geometry [56]. The intense CD signals of **51**·(l-LeuOEt)_2_ complexes were diagnostic of guest absolute configuration as the accommodation of bulky guests (e.g., LeuOEt) results in induction of strong positive bisignate Cotton effect in the Soret region. The positive Cotton effects induced in host-guest systems indicate that the tweezer adapts a clockwise twist. In such conformation, the aminoester side chain faces outside of the intraporphyrin cavity—the opposite conformer would not be favoured for steric reasons (Figure 13).

**Figure 14:**
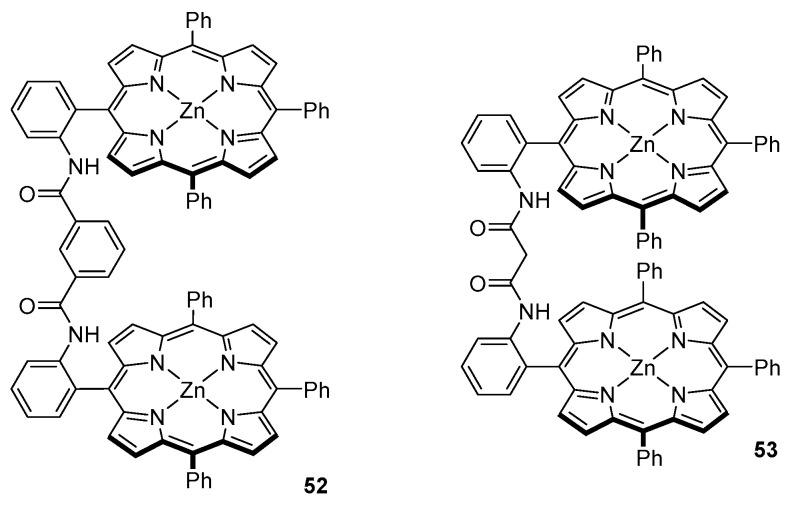
*Bis*-porphyrin tweezers with *m*-phthalic diamide and malonamide linkers.

In the efforts to provide a more sensitive chirality probe, which would accommodate chiral ligands with higher CD amplitude values, the group designed a *bis*-porphyrin tweezer with slightly less rigid linker, that is *m*-phthalic diamide (Figure 14, **52**).

The major feature of this tweezer is its aggregation in solution. It was found that even at concentrations as low as 10^−7^ M, an aggregated form remains in equilibrium with single molecules. The nature of such aggregates was elucidated by means of UV/Vis spectroscopy—**52** shows a Soret band at 418 nm and its shoulder at 427 nm, which increases the intensity at higher concentrations. This shoulder was assigned to a form in which Zn centres are five-coordinated, which is manifested by a bathochromic shift in the Soret region [57].

Host-guest complexes obtained with **52** and a series of nonpolar amino acid esters, including Ala, Val, Leu, Phe and Phg, exhibit bisignate Cotton effects in the Soret region with amplitudes around 10 times larger than those reported earlier for their oxalyl amide linker [47,56]. The signs of CD responses obtained for guests of the same handedness were also opposite to those reported previously. It was concluded that different mechanisms govern formation of host-guest complexes of those two sensors. Indeed, based on a comparative study with an aminoalcohol (α-leucinol), it was found that, in **52**, hydrogen bonds are formed between linker NH as a donor and guest carbonyl oxygen as an acceptor.

Similar results were obtained for an even more flexible malonamide-linked *bis*-porphyrin tweezer **53** (Figure 14) [58]. Host-guest complexes of this sensor and nonpolar amino acid ethyl esters exhibit CD features closely related to those obtained with a *m*-phthalic diamide linked tweezer, namely the sign of induced Cotton effects and their amplitude. Positive sign of induced Cotton effects indicates that tweezers **52** and **53** adapt into a right-handed screw. Hence, it was concluded that chiral recognition is governed by the same principles in those two types of chirality sensors [49,58].

**Figure 15:**
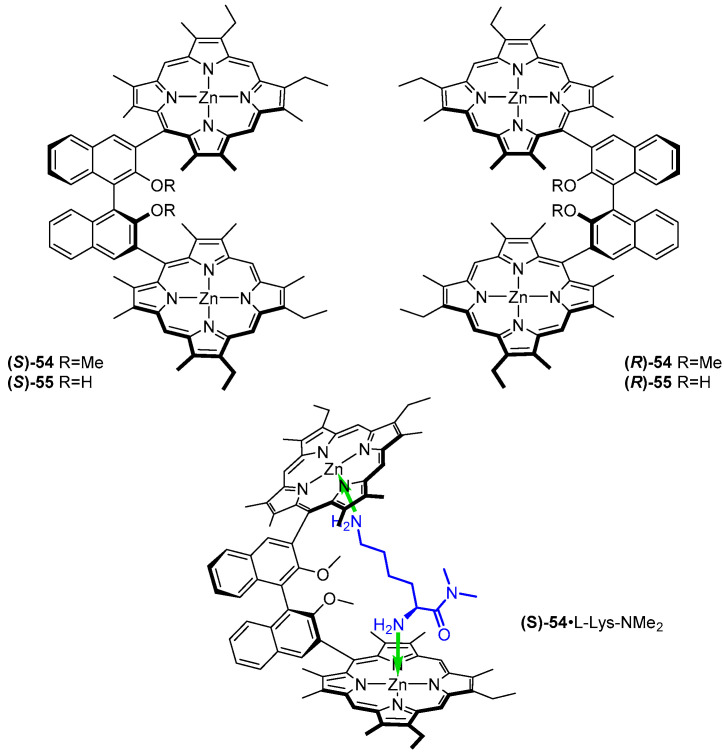
Binaphthyl-bridged porphyrin tweezers as sensors for lysine derivatives.

**Figure 16:**
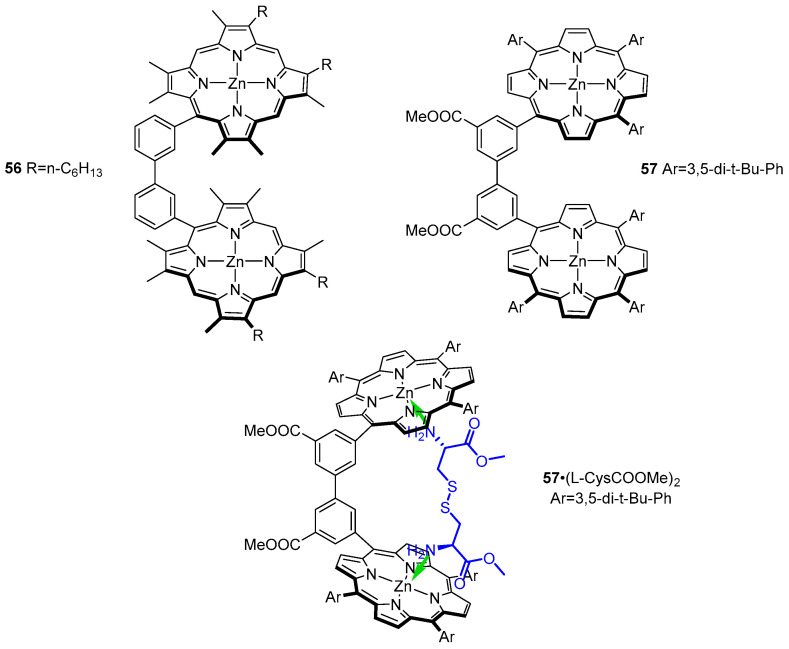
Biphenyl-bridged porphyrin tweezers as sensors for cysteine diesters.

A set of significantly more rigid *bis*-porphyrin tweezers was reported by Ogoshi and co-workers (Figure 15 and Figure 16) [59]. Porphyrin subunits were connected via chiral 2,2′-dihydroxy- (**54**) and 2,2′-dimethoxy-1,1′-binaphthyl (**55**) as well as achiral 3,3′-biphenyl (**56**) and 5,5′-dimethoxycarbonyl- 3,3′-biphenyl (**57**) bridges. Such sensors were applied in chiral recognition of lysine derivatives (Figure 15) and cystine diesters (Figure 16), manifested by induction of strong CD response in the Soret region of the host. The bisignate Cotton effects were diagnostic of guest absolute configuration. Association constants for formation of 1:1 complexes were determined by means of UV/Vis spectroscopic titration and remained in the ranges of 1.1 × 10^4^–1.6 × 10^5^ and 9.0 × 10^4^–2.4 × 10^6^ M^−1^, for binaphthyl- and biphenyl-bridged hosts, respectively.

Binaphthyl-bridged tweezers **54** and **55** show remarkable enantioselectivity for lysine derivatives, reaching 12 for d/l-Lys-NMe_2_, **(*S*)-54** favouring its l isomer (11.5) and **(*R*)-54** its d isomer. It was found that the methoxy groups in the cavity of **54** play a crucial role in chiral differentiation, as corresponding sensor **55** exhibited negligible enantioselectivity towards the same set of guests.

**Figure 17:**
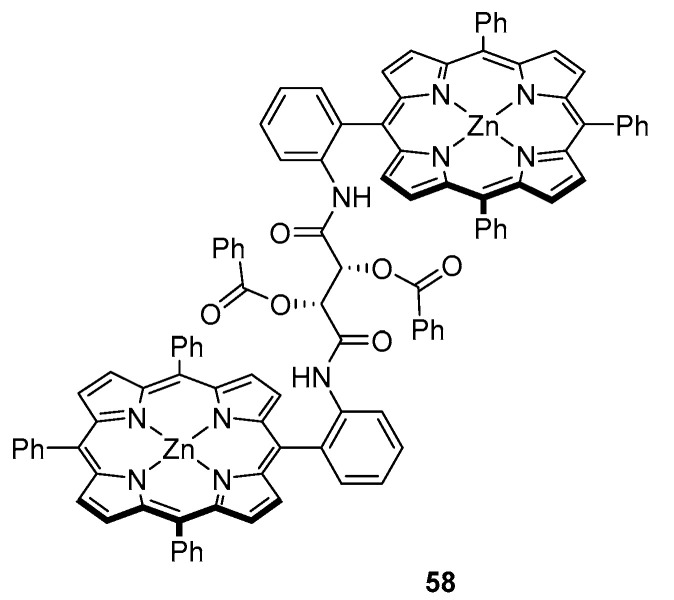
Porphyrin tweezer with tartaric acid-based linker.

Another example of chiral hosts was recently reported by Wang et al. [60], who used a fairly flexible tartaric acid-based linker in the design of their sensor for nonpolar amino acid ethyl esters (Figure 17). Compound **58** was found to form 1:2 complexes with both l and d isomers, with K_1_ in the range of 4.3 × 10^3^–6.8 × 10^4^ M^−1^ and K_2_: 6.6 × 10^2^–3.0 × 10^3^ M^−1^. Notably, while initial formation of 1:1 complexes seems to be equally easy as in the case of oxalic amide-linked tweezer reported by Hu et al. [47,56], association constants determined for subsequent coordination of another guest molecule significantly lower in this case. Formation of diastereoisomeric host-guest complexes results in a noticeable bathochromic shift, from 419 nm for **58** to 428 nm for **58**·L_2_.

This chiral sensor shows a negative CD couplet at 416 and 425 nm; coordination of l-amino acid ethyl esters gives rise to a new negative Cotton effect at 426–428, 434 and 436 nm, while their chiral counterparts induce a positive Cotton effect at 424–426 and 430–433 nm. Compound **58** was reported to show enantioselectivity towards d-aminoesters, reaching 8.4, which, according to the authors, is the highest value obtained to date for this class of sensors. Optimised structure of **58** revealed two types of hydrogen bonds—both between linker amino and guest carbonyl groups and the other way around. Peptoids constitute a class of peptidomimetics, with the amino acid side chain attached to the nitrogen instead of the α-carbon. In other words, a peptoid is a synthetic *N*-substituted glycine oligomer [61]. Lately, several porphyrin–peptoid conjugates have been designed as energy transfer systems [62]. These hybrids have been of particular interest owing to the simplicity of backbone modification, which is a powerful tool to control the change in inter-chromophore interactions [63,64].

**Figure 18:**
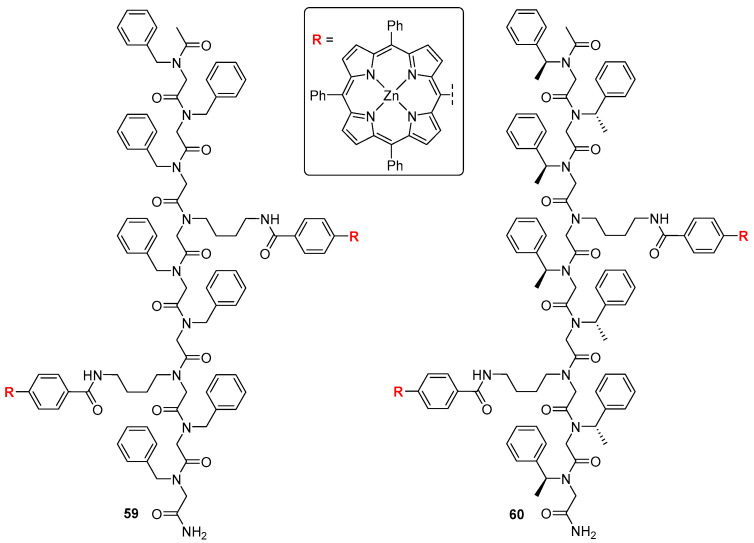
Porphyrin tweezers with achiral and chiral peptoid linkers.

Recently, Seo et al. [65] reported *bis*-porphyrin tweezers with chiral and achiral peptoids as flexible linkers (Figure 18). Those tweezers were shown to accommodate achiral diamines of various sizes and basicities, but also served the purpose of chiral recognition of lysine stereoisomers, which was monitored with UV/Vis and CD spectroscopies. This bidentate ligand was found to bind to achiral host **59**, giving rise to a bisignate Cotton effect in the Soret region (at 425 and 432 nm) reflecting guest absolute configuration. Coordination with l-LysOMe induced a negative Cotton effect, indicating that the complex formed a left-handed screw, while—as expected—d-LysOMe gave a mirror CD image, with a right-handed screw.

When a chiral tweezer **60** was employed, accommodation of chiral guests led to diastereoisomeric supramolecular structures. Upon CD monitored titration of **60** with d-LysOMe, a slight bathochromic shift of the spectrum was observed, from 422 and 430 nm to 425 and 435 nm. A complex with l isomer gave a diminished “M”-shaped CD response (425, 430 and 435 nm), probably owing to the presence of multiple CD-active conformers in the solution.

### 3.3. Aqueous Phase

Studies on chiral recognition of biomolecules offer insight into corresponding host-guest phenomena in biological systems. Therefore, investigation of such interactions in water has been of particular interest, as mimicking physiological conditions provides more precise information useful to elucidate specific mechanisms governing chiral recognition in vivo. The vast amount of porphyrin receptors has brought to light some of the most important factors influencing the process of chiral recognition of amino acids in nonpolar solvents, however the environment affects those interactions in a non-negligible way. In organic solutions, hydrogen bonding has been found to play a crucial role in two- (or three-)point fixation of guest molecule onto the porphyrin plane, yet it is considerably weakened in water due hydration [66]. In this paragraph we report some of the recent water-soluble porphyrin sensors for chiral recognition of amino acids and elucidation of factors influencing such interactions in aqueous media.

**Figure 19:**
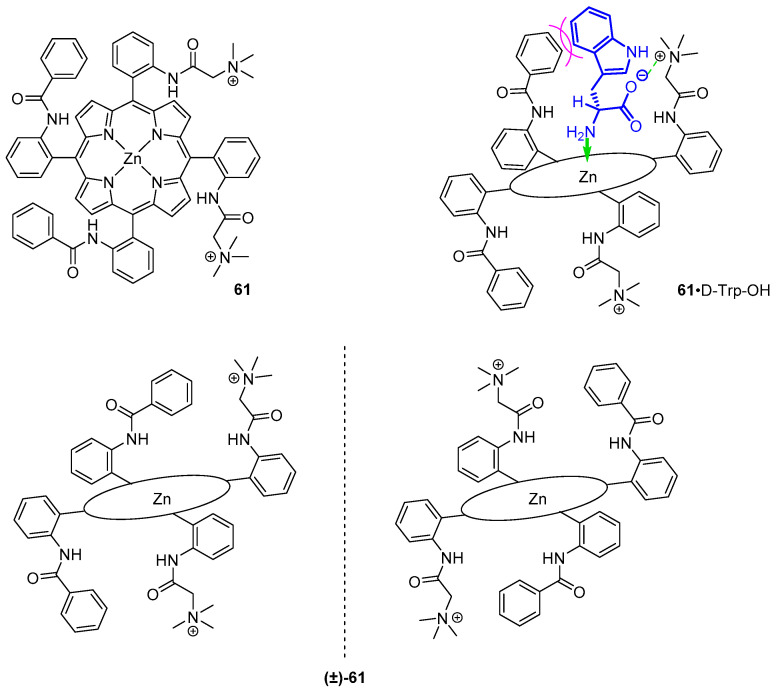
Ionic porphyrin probes for detection of amino acids.

Among early reports is a water-soluble porphyrin host for chiral recognition of amino acids reported by Imai et al. [11,67]. Their sensor bears two quaternary ammonium groups above and below porphyrin plane, bound to adjacent *meso*-phenyl substituents (Figure 19). At the two remaining *meso*-phenyls, benzamide moieties were embedded for host-guest hydrophobic attractions. Enantiomers of **60** were tested for binding amino acids in water solutions at basic pH values, where their carboxylic group is deprotonated. In this study, charged (Ser, Asp and Glu) and nonpolar (Gly, Ala, Val, Leu, Phe and Trp) amino carboxylates were compared, together with di- and tripeptides (GG, GA, GV, GL, GF, GW, GP, GS, GE, GGG and GGW). Notably, association constants for formation of such host-guest complexes in aqueous solutions are 1–3-fold lower than those reported for organic-phase systems [47] and were in the range of 2.4 × 10^1^–1.5 × 10^3^ M^−1^. In aqueous solution, guest molecules compete with H_2_O coordinated to the zinc centre. Compound **61** showed slight enantioselectivity towards enantiopure ligands, ranging from 1.2 to 3.3.

Coordination of guest NH_2_ to host zinc(II) centre and Coulomb interactions between cationic *meso* substituents and deprotonated carboxy groups of the amino acids were found to contribute to the binding constant together with hydrophobic attraction between amino acid side chain and the benzamide phenyl ring or steric repulsion between them. The latter depends on the structure of amino carboxylate in the following way. Among the most tightly bound guests are tryptophan and phenylalanine; less hydrophobic residues, such as serine, aspartic acid or glycylserine, do not show an increase in association constant, yet their stereoisomers are bound to the host selectively. Dependence of association constant on ionic strength of the solution was determined, supporting the contribution of electrostatic interactions to formation of host-guest complexes (*n-*butylamine was used as reference). Recognition ability towards GX dipeptides (where X = G, A, V, L, F, W, P, S or E) was retained, even though the asymmetric centre is separated from the coordination site.

**Figure 20:**
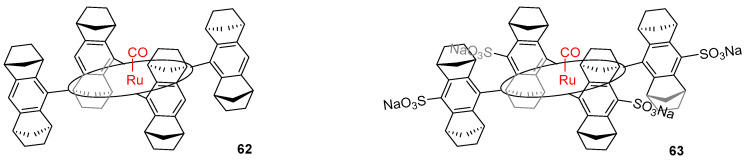
Ruthenium porphyrin complexes as amino acid sensors for organic and aqueous phase.

Chiral recognition of nonpolar amino acids by ruthenium Halterman porphyrins in chloroform, methanol and water was reported by Nicolas et al. [68]. In this study, a rigid porphyrin sensor **62** with Ru(CO) centre was employed to investigate ligation of the metal centre in *trans* position to the other axial ligand (Figure 20).

The application of a chiral host for recognition enabled the differentiation and quantification of diastereoisomeric complexes formed upon addition of racemic ligands in ^1^H NMR. Among the chiral amines investigated were alanine, phenylalanine and valine as acids (as guests for **63** in aqueous phase) and methyl esters (for **62** in organic phase studies). In all environments, d-phenylalanine was favoured over its enantiomeric counterpart, while Val and Ala showed the opposite preference. In the case of phenylalanine, a drastic decrease of enantioselectivity was observed while changing the environment from organic to aqueous; it was therefore concluded that hydrophobic interactions play a significant but not crucial role in the process of chiral recognition.

Based on previous studies on the origin of chiral recognition and its dependence on different association and dissociation constants in the formed complexes [69,70], the latter were examined with saturation transfer NMR technique, exploiting the slow axial ligand exchange in ruthenium porphyrins [45]. The data obtained from these experiments reveal that dissociation rate constants were lower for isomers, towards which selectivity was observed (i.e., k_L-PheOMe_ = 0.36 s^−1^ and k_D-PheOMe_= 0.14 s^−1^), indicating that the rate of ligand dissociation affects the process of chiral recognition.

## 4. Conclusions

We highlight the importance of the detection of natural derivatives, specifically focusing on amino acids, by two different molecular probes, BODIPY and porphyrins. The two probes essentially work with two different recognition approaches: BODIPY probes rely on the direct interaction with the target, hence a covalent interaction. Porphyrins probes, on the other hand, mostly interact with the target in the fashion of molecular recognition, which is mediated by non-covalent interactions—a synthesis of coordination, hydrogen bonding, hydrophobic interactions, steric repulsion and/or electrostatic attraction. To this day, sensors for both molecular recognition and chiral discrimination are designed. The development of such systems is of significant importance for the scientific community since there are already many specific applications, especially in the fields of biomedicine and molecular devices.
